# Coasting effect of *n*-hexane-induced neuropathy evidenced by electroneuromyography and clinical symptom scales: a 12-month follow-up study

**DOI:** 10.2478/aiht-2026-77-4034

**Published:** 2026-03-30

**Authors:** Yusuf Koçak, Uğur Kulu, Tuba Demirer Çeker, Orhan Sümbül, Dürdane Aksoy, Betül Çevik

**Affiliations:** Tokat Gaziosmanpaşa University Faculty of Medicine, Department of Neurology, Tokat, Turkey

**Keywords:** electroneuromyography, Functional Independence Measure, motor conduction velocities, muscle weakness, numbness, Numeric Pain Rating Scale, Overall Neuropathy Limitations Scale, pain severity, Self-Leeds Assessment of Neuropathic Symptoms and Signs, sensory disturbances, brzina provodljivosti motoričkih živaca, elektroneuromiografija, mišićna slabost, mjera funkcionalne neovisnosti (FIM), numerička ljestvica procjene bola (NPRS), osjetilni poremećaji, samoprocjena neuropatskih simptoma i znakova (S-LANSS), težina bola, ukupna ljestvica ograničenja kod neuropatije (ONLS), utrnulost

## Abstract

To the best of our knowledge, no study so far has investigated the coasting effect of neuropathy caused by occupational exposure to *n*-hexane through more detailed clinical evaluations than nerve conduction measurements, such that would also include pain assessment and functional status. The aim of our study was therefore to see if our measurements, which include all three elements, would support the coasting effect and reveal associations between electrophysiological measurements and clinical symptom assessments over a 12-month follow-up. Our study included eighteen patients working in the same shoe factory who were diagnosed with occupational neuropathy most likely caused by exposure to *n*-hexane. After identification of occupational exposure to high, yet unspecified VOC levels by local health authorities, production was suspended and all patients removed from further exposure. All underwent detailed neurological examination, including electroneuromyography (EMG) and the assessment of self-rated pain severity, symptoms, and functionality in everyday activities. All clinical, laboratory, and electrophysiological assessments were performed at baseline and at months 4 and 12 of follow-up. The patients presented with different degrees of numbness, muscle weakness, and sensory disturbances. EMG measurements and clinical scales at month 4 revealed significant worsening from the baseline (p<0.05), and the assessments at month 12 a significant improvement from month 4 (p<0.05), which confirms the coasting effect. Significant correlations were found between baseline motor conduction velocities and subsequent functional outcomes, and between selected sensory parameters and pain severity. Regardless of its limitations, our study points to the real-life consequences of exposure to harmful VOCs in poorly controlled shoe factory environments and establishes the coasting effect through more than one diagnostic parameter. From a preventive perspective, our findings suggest that early electrophysiological evaluation, together with simple and validated clinical scales, may help identify workers at risk of developing neuropathy before irreversible functional impairments occur. Such an approach could inform timely exposure control, medical surveillance, and occupational interventions in high-risk settings.

## Abstract (hr)

Prema našim spoznajama, dosad se fenomen *coastinga* (naknadno pogoršanje nakon prestanka izloženosti) kod neuropatije prouzročene profesionalnom izloženošću *n*-heksanu utvrđivao samo mjerenjem električne provodljivosti živaca, a zanemarivale su se procjene bola i funkcionalnog statusa. Našim dvanaestomjesečnim ispitivanjem željeli smo obuhvatiti te kliničke metode i utvrditi potkrepljuju li naši nalazi fenomen *coastinga* i jesu li elektrofiziološka mjerenja i kliničke procjene simptoma povezani. Ispitivanje je obuhvatilo 18 pacijenata, zaposlenih u istoj tvornici obuće, kojima je dijagnosticirana profesionalna neuropatija, najvjerojatnije prouzročena izloženošću *n*-heksanu. Nakon što su lokalne zdravstvene vlasti utvrdile profesionalnu izloženost visokim razinama nedefiniranih hlapljivih organskih spojeva, proizvodnja je obustavljena. Svih 18 radnika bilo je podvrgnuto detaljnom neurološkom pregledu, uključujući elektroneuromiografiju (EMG) i subjektivnu procjenu razine bola, simptoma i funkcionalnosti u svakodnevnim aktivnostima. Sva klinička, laboratorijska i elektrofiziološka mjerenja provedena su na početku istraživanja te u 4. i 12. mjesecu praćenja. Pacijenti su manifestirali različite stupnjeve utrnulosti, mišićne slabosti i osjetilnih poremećaja. EMG i kliničke ljestvice u 4. mjesecu praćenja pokazali su značajno pogoršanje u odnosu na početno stanje (p<0,05), a rezultati u 12. mjesecu pokazali su značajno poboljšanje u odnosu na 4. mjesec (p<0,05), što potvrđuje fenomen *coastinga*. Utvrđene su značajne korelacije između početnih brzina motoričke provodljivosti i kasnijih funkcionalnih ishoda, kao i između odabranih osjetilnih parametara i razine bola. Bez obzira na ograničenja, naše istraživanje upozorava na stvarne posljedice izloženosti štetnim hlapljivim organskim spojevima u slabo kontroliranim okružjima poput tvornica obuće te potvrđuje fenomen *coastinga* ne temelju više dijagnostičkih parametara. U smislu prevencije, naši rezultati upućuju na to da rani elektrofiziološki nalazi, uz primjenu jednostavnih i validiranih kliničkih ljestvica, mogu pomoći prepoznati radnike s rizikom od nastanka neuropatije prije nego što dođe do nepovratnih funkcionalnih oštećenja. Takav pristup mogao bi pridonijeti pravovremenoj kontroli izloženosti, medicinskom nadzoru i profesionalnim intervencijama u visokorizičnim okružjima.

First identified in 1964, *n*-hexane is an aliphatic hydrocarbon that acts as a peripheral nerve toxin (1). It is widely used as organic solvent in industrial adhesives, cleaners, varnishes, and polishes but is also abused as an inhalant (2). Although the incidence of neuropathy induced by *n*-hexane in developed countries has fallen behind other causes of toxic neuropathy, it continues to be significant in developing countries. The main pathology is caused by its metabolites, 2,5 hexanedione and other γ-diketones, which interfere with neurofilament transport, especially in myelinated thick and long axons, in which they accumulate and cause axon swelling, especially near the nodes of Ranvier, leading to myelin shrinkage and, ultimately, to distal axonopathy (3). Although the main pathology is axonal, demyelinating features can be identified with nerve conduction measurements, because they affect the paranodal myelin sheaths, and even though thick myelinated neurons are the first to suffer, *n*-hexane toxicity has also been reported in small-fibre neuropathies (4).

Clinical manifestations of *n*-hexane-induced neuropathy may vary, depending on exposure duration and intensity. Long-term low-dose exposures usually cause a relatively slowly progressing peripheral neuropathy characterised by numbness, especially in the distal lower extremities, and muscle weakness (5). High dose exposures over a short period may present with acute-subacute severe clinical conditions that make differentiation with the Guillain-Barré syndrome difficult (6).

Like other toxic neuropathies, the one induced by *n*-hexane is characterised by the “coasting effect”, first described by Yamamura (7), a condition which deteriorates after exposure to a toxic agent has stopped but stabilises and improves over time (8,9,10,11).

One of the most important tests for clinical diagnosis and follow-up of neuropathy is electroneuromyography (EMG), which can detect amplitude drops caused by primary axonal degeneration and conduction blocks and slowdowns caused by secondary demyelination (12). However, neurological tests alone are often insufficient to assess the clinical condition. For example, two patients with similar sensory deficits or muscle weakness may experience different pain or different ability to perform everyday activities.

To the best of our knowledge, no study has demonstrated the coasting effect through more detailed clinical evaluations that would encompass neurological examination, pain assessment, and functional status in patients presenting with *n-*hexane-induced neuropathy. The aim of our study was therefore to address this gap, see if our findings support the coasting effect, and perhaps reveal associations between EMG measurements and symptom assessments over a 12-month follow-up.

## PARTICIPANTS AND METHODS

### Participants and study design

Our study was motivated by the diagnosis of the first patient who presented with severe symptoms encountered in acute case reports (5). She developed respiratory distress and cranial nerve involvement during follow-up. At first, we considered the Guillain-Barré syndrome and started intravenous immunoglobulin (IVIG) treatment for tetraparesis, areflexia, loss of F responses on EMG (sural nerve conduction was normal) but discarded this diagnosis as the patient was unresponsive to IVIG treatment. In a short time, other patients with similar symptoms were admitted to the hospital, including the one who worked at the same workplace and in a closely related unit and was suspected of exposure to an *n*-hexane-containing solvent-based adhesive. Their neurological and electrophysiological findings were in line with toxic neuropathy described in the literature and the patients were diagnosed in accordance with the algorithms proposed by Berger and Schaumburg (13) and London et al. (14). All patients later included in this study were tested for a toxicology panel, especially blood and urine 2,5-hexanedione, but showed no positive findings. We attributed this to the fact that the patients left the workplace with the onset of very mild symptoms, and the samples were sent approximately 14 days after the exposure to the toxic agent had stopped. We did not find it surprising, considering that the half-life of *n*-hexane metabolites is 2–3 h in blood, 15 h in urine, and 64 h in adipose tissue (15).

Driven by our suspicion, however, we started this study by enrolling 29 patients working in the same shoe factory, who were admitted to the Tokat Gaziosmanpaşa University Education and Research Hospital between March and July 2023 over acute toxic polyneuropathy, established using the clinical and electrophysiological diagnostic algorithm proposed by Mirian et al. (16).

All study participants were employed in a small-scale shoe factory. The adhesive agent used in the production was Denlaks YD-6476™ (Oynurden Chemicals, Istanbul, Turkey). After the onset of poisoning, an official inspection was conducted by the Provincial Directorate of Health and the Provincial Directorate of Disaster and Emergency Management. The environmental analysis revealed an unspecified volatile organic compound (VOC) mixture in the concentration of 385 ppm (1,000–1,500 mg/m^3^). As a result, production was suspended and legal proceedings initiated, and since the judicial process is still ongoing, no further information has been made available to us.

**Figure 1:**
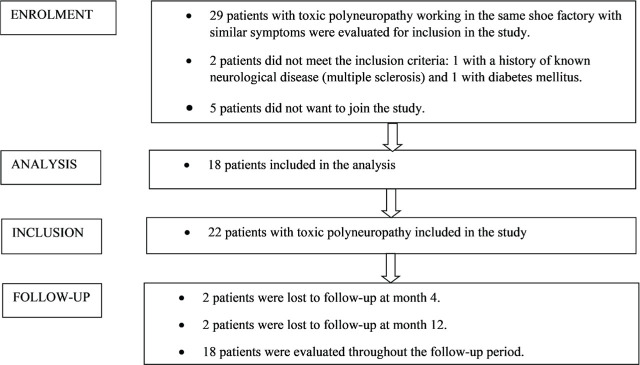
Participant selection for inclusion in the study

Eleven patients were excluded because they had a history of known neurological disorder (e.g., multiple sclerosis, myasthenia gravis, and muscular dystrophy), comorbidities (e.g., diabetes mellitus and thyroid diseases), or alcohol or other substance abuse that might cause neuropathy, because their clinical records were missing relevant data, or because they dropped out (Figure 1). The study was completed with 18 patients.

All patients, regardless of the inclusion/exclusion criteria, were referred to the Department of Physical Medicine and Rehabilitation and received tailored home exercise treatment to address their neurological deficits.

Ethical approval for the study was obtained from the Tokat Gaziosmanpaşa University Clinical Research Hospital ethics committee (approval No. 24-KAEK-209), and the study was conducted in line with the Declaration of Helsinki. All participants gave informed consent to participation.

We first collected demographic information, including age, gender, body mass index (BMI), history of known diseases and taking medicines, years of work at the shoe factory, and operational unit (workplace). All participants then underwent a detailed neurological examination that included baseline electroneuromyography (EMG) and self-reported assessments, namely, the Numeric Pain Rating Scale (NPRS), Self-Leeds Assessment of Neuropathic Symptoms, Signs (S-LANSS) pain scale, Functional Independence Measure (FIM), and Overall Neuropathy Limitations Scale (ONLS). We also ran blood and urine tests. All clinical, laboratory, and electrophysiological assessments were performed three times: at baseline, at month 4 (after three full months passed), and during the 12^th^ month (month 12) of follow-up.

### Electrophysiological measurements

Following the standard polyneuropathy electrodiagnostic protocols (17), we used a Nihon Kohden MEB-9600 EMG model (Nihon Kohden Corporation, Tokyo, Japan) to measure sensory and/or motor nerve conduction in all participants, including the right median nerve motor distal latency (rMMDL), right median nerve motor amplitude (rMMAmp), right median nerve motor velocity (rMMVel), right median nerve sensory distal latency (rMSDL), right median nerve sensory amplitude (rMSAmp), right median nerve sensory velocity (rMSVel), right ulnar nerve motor distal latency (rUMDL), right ulnar nerve motor amplitude (rUMAmp), right ulnar nerve motor velocity (rUMVel), right ulnar nerve sensory distal latency (rUSDL), right ulnar nerve sensory amplitude (rUSAmp), right ulnar nerve sensory velocity (rUSVel), right common peroneal nerve motor distal latency (rCPMDL), right common peroneal nerve motor amplitude (rCPMAmp), right common peroneal nerve motor velocity (rCPMVel), right tibial posterior nerve motor distal latency (rTPMDL), right tibial posterior nerve motor amplitude (rTPMAmp), right tibial posterior nerve motor velocity (rTPMVel), right sural nerve sensory distal latency (rSSDL), right sural nerve sensory amplitude (rSSAmp), right sural nerve sensory velocity (rSSVel), left sural nerve sensory distal latency (lSSDL), left sural nerve sensory amplitude (lSSAmp), left sural nerve sensory velocity (lSSVel), the F wave of the right common peroneal nerve (FrCP), F wave of the right tibial posterior nerve (FrTP), F wave of the right median nerve (FrM), and the F wave of the right ulnar nerve (FrU).

All measurements were performed by the same practitioner. The room and limb temperature were kept above 30 °C throughout all measurements.

### Numeric Pain Rating Scale

To assess pain severity in our participants, we relied on the self-reported NPRS, a one-dimensional version of the visual analogue scale (VAS) described in detail elsewhere (18). The participants were asked to rate the severity of their pain over the past week on a 11-point scale from zero (no pain) to 10 (the most severe pain they could imagine). To assess changes in pain severity, we relied on the recommendations issued by the Initiative on Methods, Measurement, and Pain Assessment in Clinical Trials (IMMPACT) (19), whereby a ≥30 % (or a ≥2-point) decrease in the NPRS score represents minimal clinically important difference (MCID), and a ≥50 % decrease a substantial improvement (20).

### Self-Leeds Assessment of Neuropathic Symptoms and Signs

To assess whether the reported pain was of neuropathic origin we asked the participants to complete the 7-item self-reported S-LANSS scale ranging from 0 to 24 (21). It consists of five symptom items and two self-examination items. Symptom items include questions about needling, skin colour changes, increased skin sensitivity, “electric shock” type of pain, and “burning pain”. The two self-examination items also include allodynia and numbness. The standard diagnostic cut-off is ≥12 points, indicating a high probability that pain has a neuropathic origin. This threshold has been validated in multiple languages and clinical contexts (22,23).

### Functional Independence Measure

To assess the degree of functional independence in everyday activities of our participants, we relied on the 18-item FIM questionnaire, which measures motor and cognitive functional independence (24). Each item consists of a 7-point scale ranging from 1 (total dependence) to 7 (total independence).

The motor function section consists of self-care, sphincter, transfer, and locomotion sub-sections, and the total score ranges from 13 to 91. Patients scoring between 13 and 37 points are classified as having severe dependence, which means that they require maximal help for most daily motor activities. Scores between 38 and 64 points indicate moderate dependence, that is, a need for partial help in mobility and self-care. Those who score between 65 and 91 points are considered functionally independent or recovered, as they can perform most daily activities with no or only minimal help (25,26,27).

The cognitive function section consists of communication and social cognition sub-sections, each ranging from 5 to 35 points. Total FIM score ranges from 18 to 126, and high scores indicate patient’s cognitive independence. However, as all participants presented with peripheral toxic neuropathy without any central nervous system involvement or cognitive impairment, we limited our assessment to motor independence and mobility, which are directly affected by peripheral nerve damage.

### Overall Neuropathy Limitations Scale

The ONLS scale evaluates the upper and lower extremities separately, and the total score (12 points) is obtained by summing the upper (maximum 5 points) and lower extremity (maximum 7 points) scores. Higher scores indicate higher disability. The first question is about the presence of sensory symptoms, such as tingling, numbness or weakness in the upper extremities, while the rest seeks to establish functions such as washing/combing hair, locking a door with a key, using cutlery, unbuttoning buttons and zippers, putting on clothes, difficulties climbing and running on stairs or walking, abnormal gait, walking with single or double support, moving with a wheelchair, getting up from a wheelchair, standing and walking 10 metres, and using an ankle orthosis or a brace (28).

The most widely accepted MCID for ONLS is a change of ≥1 point in the total score, indicating a clinically meaningful improvement or deterioration. Unlike broader functional scales such as the FIM, the ONLS does not categorise severity (e.g., mild, moderate, or severe disability). Instead, it is an ordinal scale in which each one-point change represents a clinically meaningful stepwise difference, interpreted in the clinical context rather than against predefined universal thresholds (29,30,31).

### Laboratory analysis

All patients underwent complete blood count, glucose, vitamin B12, HbA1c, serum electrolytes, and kidney, liver, and thyroid function tests. In addition to routine blood testing performed in our hospital, the participant’s blood and urine samples were analysed at the Gazi Mustafa Kemal Occupational and Environmental Diseases Hospital in Ankara, which is the country’s reference centre for detailed toxicological analyses and which ran tests for 2,5 hexanedione and hippuric acid, typical toxic agents encountered in shoe factories.

### Statistical analysis

Data are expressed as means and standard deviations (SD). Given the small sample size and the non-normal distribution, as confirmed by the Shapiro-Wilk test, non-parametric tests were deemed appropriate for analysis. Differences between independent variables were evaluated using the Mann-Whitney *U* test. The Friedman test evaluated differences between clinical scale results against each EMG parameter. For parameters which showed a significant difference finding (p<0.05), we ran post-hoc analysis using pairwise Wilcoxon signed-rank tests with a Bonferroni correction for multiple comparisons.

Spearman’s correlation was used to compare the baseline nerve conduction results with follow-up measurements of the clinical scales at month 4 for a possible prognosis (ϱ: Spearman’s correlation coefficient of r<0.4 was considered weak, r=0.4–0.6 moderate, and r=0.6–1.0 high correlation).

Then we divided the baseline nerve conduction velocities into normal and pathological according to laboratory standards and compared mean values of the clinical scales with nerve conduction velocities to obtain a statistical relationship between the two for possible prognosis. P values of <0.05 were considered statistically significant. All statistics were run on IBM SPSS Statistics 22 (IBM, Armonk, NY, USA).

To determine the statistical power achieved in this study we ran a post-hoc power analysis using the G*Power software version 3.1.9.7 (Heinrich Heine University, Düsseldorf, Germany). Given the sample size of 18, alpha level of 0.05, and the observed effect size of 0.910 for NPRS, the analysis revealed the statistical power of 0.99. This indicates that the study had a 99 % chance of detecting the observed effect, which is well above the conventional threshold of 0.80. Besides the above alpha level of 0.05 and sample size of 18, the observed effect size of 0.700 for S-LANSS rendered the statistical power of 0.89. For FIM and ONLS the observed effect size was 1.00 and the statistical power was 0.99.

## RESULTS

Table 1 shows the baseline participant demographic, body mass index (BMI), and work data. According to the information provided by the participants, the operational units were not separated by walls to contain exposure. Instead, all units operated side by side in a large common hall. None of the patients had any known chronic or systemic diseases. None were taking regular medication. There was no statistically significant correlation between the duration of employment and clinical severity or electrophysiological findings.

**Table 1:** Participant demographic and occupational data

**Age (years) (mean ± SD)**	
Total (n=18)	30.05±12.66
Women (n=14)	30.37±13.38
Men (n=4)	28.75±10.84
**Body mass index (kg/m^2^)**	24.99±4.06
Operational units^*^ (n=18)	
lasting/assembly unit	9
stitching/closing unit	7
primer coating unit	2
**Duration of employment at the shoe factory (months) (mean ± SD)**	27.55±19.54

* The lasting/assembly unit mainly involves sole attachment using solvent-based adhesives; the stitching/closing unit includes mechanical sewing of the upper parts, while the primer coating unit is responsible for surface preparation using primer solutions prior to bonding. SD – standard deviation

In all 18 patients, the first neurological symptom was numbness in the legs. The most common symptoms on hospital admission were numbness in the hands and feet. The second most common symptom was difficulty walking. In 10 patients, muscle weakness started simultaneously with numbness, and four of them were hospitalised because of severe muscle weakness. Respiratory distress and cranial nerve involvement developed in one patient. All patients reported weight and appetite loss and fatigue as accompanying symptoms.

No mental disorders were found in any. Glove-sock style hypoesthesia was detected in all patients. The vibration sense test revealed impairment in 12 patients at baseline, which affected all patients by the end of the study (follow-up at months 4 and 12). The Achilles reflex was decreased in all patients at baseline. In the follow-up examinations, a significant decrease was observed in other reflexes (patella, biceps, brachioradialis, and triceps). No other neurological pathology was detected in any patient either at baseline or during the follow-up.

Table 2 shows that participants’ NPRS, S-LANSS, FIM, and ONLS scores at month 4 of follow-up worsened and at month 12 improved significantly compared to baseline (p<0.05), which points to the “coasting effect” of toxic neuropathy.

**Table 2:** Changes in clinical scale scores from baseline to month 12 of follow-up

	**Baseline**	**4^th^ Month**	**12^th^ Month**	**p**
NPRS	7.80±1.54 *^a^*	8.75±1.29 *^b^*	4.50±2.89 *^c^*	<0.001^*^
S-LANSS	15.25±5.75 *^a^*	18.35±4.50 *^b^*	6.75±9.42 *^c^*	<0.001^*^
FIM	48.85±14.97 *^a^*	36.55±17.83 *^b^*	73.60±10.04 *^c^*	<0.001^*^
ONLS	5.10±2.36 *^a^*	8.40±3.39 *^b^*	2.60±1.73 *^c^*	<0.001^*^

* statistically significant differences are marked with different letters in superscript (Friedman test, followed by Bonferroni correction for multiple comparisons). FIM (Functional Independence Measure), NPRS (Numeric Pain Rating Scale), ONLS (Overall Neuropathy Limitations Scale) and S-LANSS (Self-Leeds Assessment of Neuropathic Symptoms and Signs Pain Scale)

Table 3 shows changes in EMG findings from baseline to months 4 and 12. At baseline, the EMG showed a slowing of the right median nerve motor conduction velocity, right ulnar nerve motor conduction velocity, right common peroneal nerve motor conduction velocity, and bilateral sural nerve sensory conduction velocity, which did not reach the demyelinating limits but was below normal laboratory values. Nine patients had no right peroneal nerve F response, indicating proximal demyelination. On months 4 and 12 of the follow-up, EMGs showed mixed-type polyneuropathy with demyelinating and axonal degeneration in all nerves.

**Table 3:** Changes in measured electroneuromyography parameters from baseline to month 12 of follow-up

	**Reference intervals**	**Baseline**	**4^th^ Month**	**12^th^ Month**	**p**
rMMDL	3.8 ms	3.75±0.42^a^	4.48±2.43^b^	4.6±1.25^ab^	0.040^*^
rMMAmp	4.3 mV	12.28±3.49^a^	3.81±4.43^b^	6.57±4.61^c^	<0.001^*^
rMMVel	49.7 m/s	47.27±6.38^a^	37.11±20.61^b^	51.34±8.6^a^	0.026^*^
rMSDL	3.3 ms	2.93±0.36	3.18±0.49	1.95±1.78	0.252
rMSAmp	10 µV	21.18±5.52^a^	8.4±8.98^b^	17.73±4.94^c^	<0.001^*^
rMSVel	39.4 m/s	41.57±7.25^a^	21.77±18.7^b^	38.88±5.27^a^	<0.001^*^
rUMDL	3.3 ms	2.96±0.28^a^	4.13±2.25^b^	3.55±1.29^c^	<0.001^*^
rUMAmp	7 mV	13.38±2.67^a^	4.91±4.92^b^	8.73±4.12^c^	<0.001^*^
rUMVel	49.9 m/s	49.48±8.11^a^	39.41±18.18^b^	51.24±7.59^a^	0.008^*^
rUSDL	3.1 ms	2.74±0.35	2.92±0.44	1.74±1.68	0.397
rUSAmp	7 µV	20.42±4.98^a^	6.8±8.48^b^	16.56±6.34^a^	<0.001^*^
rUSVel	37.3 m/s	42.59±5.23^a^	21.14±20.11^b^	38.89±3.64^c^	<0.001^*^
rCPMDL	5.8 ms	4.79±0.76^a^	7.29±2.93	5.86±3.6	0.040^*^
rCPMAmp	3.6 mV	6.85±2.28^a^	1.58±2.8^b^	2.49±3.46^b^	<0.001^*^
rCPMVel	40.9 m/s	40.73±5.03^a^	17.51±20.64^b^	24.56±21.15^b^	<0.001^*^
rTPMDL	5.8 ms	4.49±0.69	6.69±3.24	5.41±2.23	0.285
rTPMAmp	3.6 mV	18.79±7.29^a^	3.52±5.83^b^	6.13±7.36^c^	<0.001^*^
rTPMVel	39.6 m/s	44.07±3.5^a^	22.3±21.42^b^	30.06±19.01^b^	<0.001^*^
rSSDL	3.6 ms	3.24±1	3.33±1.54	2.34±2.28	0.495
rSSAmp	5 µV	17.5±7.17^a^	5.12±6.13^b^	9.19±6.67^c^	<0.001^*^
rSSVel	40 m/s	35.27±8.89^a^	17.09±16.24^b^	28±13.54^a^	<0.001^*^
lSSDL	3.6 ms	3.55±1.05	3.71±1.71	2.56±2.43	0.495
lSSAmp	5 µV	15.59±6.06^a^	5.45±6.49^b^	9.24±6.1^c^	<0.001^*^
lSSVel	40 m/s	35.01±9.21^a^	16.56±15.97^b^	27.37±12.62^c^	<0.001^*^
FrCP	52 m/s	51.53±7.82^a^	57.39±10.76^b^	49.99±9.25^a^	0.011^*^
FrTP	52 m/s	52.18±6.09^a^	56.28±8.34^a^	50.08±7.53^a^	0.001^*^
FrM	32 m/s	30.74±4.96	33.44±7.33	30.91±6.37	0.051
FrU	32 m/s	28.23±4.55^a^	31.26±4.58^b^	29.04±6.32^a^	0.002^*^

* statistically significant differences are marked with different letters in superscript (Friedman test, followed by Bonferroni correction for multiple comparisons). FrCP – F wave of right common peroneal nerve; FrM – F wave of right median nerve; FrTP – F wave of right tibial posterior nerve; FrU – F wave of right ulnar nerve; lSSAmp – left sural nerve sensory amplitude; lSSDL – left sural nerve sensory distal latency; lSSVel – left sural nerve sensory velocity; m/s – meter per second; mV – millivolt; ms – millisecond; µV – microvolt; rCPMAmp – right common peroneal nerve motor amplitude; rCPMDL – right common peroneal nerve motor distal latency; rCPMVel – right common peroneal nerve motor velocity; rMMAmp – right median nerve motor amplitude; rMMDL – right median nerve motor distal latency; rMMVel – right median nerve motor velocity; rMSAmp – right median nerve sensory amplitude; rMSDL – right median nerve sensory distal latency; rMSVel – right median nerve sensory velocity; rSSAmp – right sural nerve sensory amplitude; rSSDL – right sural nerve sensory distal latency; rSSVel – right sural nerve sensory velocity; rTPMAmp – right tibial posterior nerve motor amplitude; rTPMDL – right tibial posterior nerve motor distal latency; rTPMVel – right tibial posterior nerve motor velocity; rUMAmp – right ulnar nerve motor amplitude; rUMDL – right ulnar nerve motor distal latency; rUMVel – right ulnar nerve motor velocity; rUSAmp – right ulnar nerve sensory amplitude; rUSDL – right ulnar nerve sensory distal latency; rUSVel – right ulnar nerve sensory velocity

Although no statistically significant difference was found in distal latencies, other EMG parameters showed statistically significant worsening at month 4 and a partial improvement at month 12 (p<0.05), which confirmed the coasting effect indicated by the clinical scales.

To assess the prognostic relevance of EMG parameters, we ran Spearman’s correlation analysis between baseline EMG measurements and clinical scale scores obtained at month 4, when clinical impairment was the most evident (Table 4). Significant correlations were predominantly observed between functional outcomes and baseline motor conduction velocities, whereas pain severity mainly correlated with sensory parameters. Specifically, FIM scores at month 4 showed significant positive correlations with baseline rMMVel, rUMVel, and rCPMVel, while ONLS scores correlated negatively with rMMVel and rUMVel. In addition, NPRS at month 4 showed a significant negative correlation with baseline rUSDL and significant positive correlation with baseline rUSVel. In addition, an isolated significant correlation was observed between FIM scores at month 4 and baseline rCPMDL. However, distal latency measures did not show a consistent pattern across the nerves.

**Table 4:** Correlations between baseline electroneuromyography parameters and clinical scale scores at month 4 of follow-up

**Baseline EMG parameters**	**Month 4**				
		**NPRS**	**S-LANSS**	**FIM**	**ONLS**
rMMDL	ϱ	−0.044	0.108	−0.380	0.325
	p	0.855	0.649	0.098	0.162
rMMAmp	ϱ	0.254	−0.326	−0.029	−0.164
	p	0.28	0.161	0.902	0.49
rMMVel	ϱ	−0.117	0.040	0.618^*^	−0.675^*^
	p	0.623	0.869	0.004	0.001
rMSDL	ϱ	−0.037	−0.042	−0.177	0.001
	p	0.878	0.861	0.454	0.997
rMSAmp	ϱ	−0.079	−0.083	0.289	−0.173
	p	0.742	0.729	0.217	0.466
rMSVel	ϱ	0.054	−0.266	0.078	−0.033
	p	0.820	0.257	0.745	0.891
rUMDL	ϱ	−0.251	−0.068	−0.047	0.309
	p	0.286	0.776	0.844	0.186
rUMAmp	ϱ	0.221	0.071	−0.358	0.205
	p	0.349	0.767	0.121	0.386
rUMVel	ϱ	0.091	−0.013	0.641^*^	−0.448^*^
	p	0.703	0.955	0.002	0.048
rUSDL	ϱ	−0.719^*^	−0.190	0.169	−0.061
	p	<0.001	0.422	0.478	0.799
rUSAmp	ϱ	0.011	−0.05	0.012	−0.075
	p	0.964	0.835	0.961	0.754
rUSVel	ϱ	0.498^*^	0.205	0.006	−0.097
	p	0.025	0.386	0.981	0.685
rCPMDL	ϱ	0.045	0.303	−0.499^*^	0.157
	p	0.850	0.194	0.025	0.509
rCPMAmp	ϱ	−0.056	−0.031	0.415	−0.299
	p	0.814	0.897	0.069	0.200
rCPMVel	ϱ	−0.12	−0.143	0.474^*^	−0.245
	p	0.615	0.548	0.035	0.298
rTPMDL	ϱ	0.283	−0.126	−0.358	0.303
	p	0.227	0.596	0.121	0.193
rTPMAmp	ϱ	−0.312	0.170	0.400	−0.251
	p	0.181	0.473	0.080	0.286
rTPMVel	ϱ	−0.19	0.183	0.384	−0.233
	p	0.423	0.439	0.095	0.323
rSSDL	ϱ	−0.216	0.004	−0.050	0.129
	p	0.359	0.987	0.835	0.588
rSSAmp	ϱ	0.210	−0.159	−0.083	0.150
	p	0.373	0.503	0.727	0.527
rSSVel	ϱ	0.125	0.021	−0.245	0.214
	p	0.601	0.93	0.298	0.365
lSSDL	ϱ	−0.164	−0.133	0.121	−0.053
	p	0.490	0.577	0.610	0.825
lSSAmp	ϱ	−0.066	−0.23	0.061	−0.005
	p	0.781	0.330	0.799	0.982
lSSVel	ϱ	0.174	0.132	−0.431	0.416
	p	0.463	0.578	0.057	0.068
FrCP	ϱ	0.246	−0.517	−0.516	0.533
	p	0.465	0.103	0.104	0.091
FrTP	ϱ	−0.155	−0.233	−0.353	0.271
	p	0.539	0.352	0.15	0.276
FrM	ϱ	−0.096	0.021	−0.43	0.352
	p	0.688	0.929	0.058	0.128
FrU	ϱ	−0.323	−0.174	−0.386	0.368
	p	0.164	0.464	0.093	0.111

* statistically significant correlation (ϱ – Sperman’s rank correlation coefficient: ϱ<0.4 – weak correlation; ϱ=0.4–0.6 – moderate correlation; ϱ=0.6–1.0 high correlation). EMG – electroneuromyography; FIM – Functional Independence Measure; FrCP – F wave of right common peroneal nerve; FrM – F wave of right median nerve; FrTP – F wave of right tibial posterior nerve; FrU – F wave of right ulnar nerve; lSSAmp – left sural nerve sensory amplitude; lSSDL – left sural nerve sensory distal latency; lSSVel – left sural nerve sensory velocity; NPRS – Numeric Pain Rating Scale; ONLS – Overall Neuropathy Limitations Scale; rCPMAmp – right common peroneal nerve motor amplitude; rCPMDL – right common peroneal nerve motor distal latency; rCPMVel – right common peroneal nerve motor velocity; rMMAmp – right median nerve motor amplitude; rMMDL – right median nerve motor distal latency; rMMVel – right median nerve motor velocity; rMSAmp – right median nerve sensory amplitude; rMSDL – right median nerve sensory distal latency; rMSVel – right median nerve sensory velocity; rSSAmp – right sural nerve sensory amplitude; rSSDL – right sural nerve sensory distal latency; rSSVel – right sural nerve sensory velocity; rTPMAmp – right tibial posterior nerve motor amplitude; rTPMDL – right tibial posterior nerve motor distal latency; rTPMVel – right tibial posterior nerve motor velocity; rUMAmp – right ulnar nerve motor amplitude; rUMDL – right ulnar nerve motor distal latency; rUMVel – right ulnar nerve motor velocity; rUSAmp – right ulnar nerve sensory amplitude; rUSDL – right ulnar nerve sensory distal latency; rUSVel – right ulnar nerve sensory velocity; S-LANSS – Self-Leeds Assessment of Neuropathic Symptoms and Signs Pain Scale

Table 5 shows that patients with normal right ulnar nerve motor conduction velocities (n=11) had better clinical scale scores than patients with abnormal velocities (n=7), but they differed significantly only in FIM at month 4 and ONLS at months 4 and 12 of the follow-up.

**Table 5:** Comparison of clinical scale scores between patients with normal and abnormal ulnar nerve motor conduction velocities at baseline

**Clinical assessments**	**Ulnar nerve motor conduction velocities at baseline (mean ± SD)**		**p**
	**Normal (n=11)**	**Abnormal (n=7)**	
NPRS baseline	7.77±1.74	7.86±1.21	0.683
NPRS month 4	8.62±1.39	9±1.15	0.591
NPRS month 12	4.38±3.25	4.71±2.29	0.600
S-LANSS baseline	12.57±2.37	16.69±6.56	0.163
S-LANSS month 4	18.14±2.54	18.46±5.36	0.968
S-LANSS month 12	3.86±8.21	8.31±9.97	0.297
FIM baseline	53.77±14.81	39.71±11	0.068
FIM month 4	44.31±16.96	22.14±7.69	0.003^*^
FIM month 12	76.62±7.32	68±12.48	0.072
ONLS baseline	4.38±1.45	6.43±3.21	0.093
ONLS month 4	7.23±3.27	10.57±2.57	0.050^*^
ONLS month 12	1.92±1.38	3.86±1.68	0.019^*^

* statistically significant differences are marked with boldface (Mann-Whitney *U* test). FIM – Functional Independence Measure; NPRS – Numeric Pain Rating Scale; ONLS – Overall Neuropathy Limitations Scale; SD – standard deviation; S-LANSS – Self-Leeds Assessment of Neuropathic Symptoms and Signs Pain Scale

Similarly, patients with normal right peroneal nerve motor conduction velocity (n=14) had better clinical scale scores (Table 6), but the differences were significant only for ONLS scores throughout the study and for S-LANSS and FIM scores at baseline (p<0.05), indicating that higher motor conduction velocities were associated with lower neuropathic pain severity and more favourable functional outcomes.

**Table 6:** Comparison of clinical scale scores between patients with normal and abnormal peroneal nerve motor conduction velocities at baseline

**Clinical assessments**	**Peroneal nerve motor conduction velocities at baseline (mean ± SD)**		**p**
	**Normal (n=14)**	**Abnormal (n=4)**	
NPRS baseline	7.63±1.51	7.92±1.62	0.551
NPRS month 4	8.42±1.31	9.25±1.16	0.136
NPRS month 12	4±2.78	4.83±3.04	0.530
S-LANSS baseline	11.88±2.47	17.5±6.27	0.020^*^
S-LANSS month 4	18.13±2.1	18.5±5.66	0.846
S-LANSS month 12	5.38±8.73	7.67±10.13	0.553
FIM baseline	54.33±14.89	40.63±11.44	0.048^*^
FIM month 4	44.5±17.09	24.63±11.55	0.009^*^
FIM month 12	77.17±7	68.25±11.91	0.102
ONLS baseline	4.17±1.47	6.5±2.83	0.012^*^
ONLS month 4	6.83±3.07	10.75±2.43	0.016^*^
ONLS month 12	1.92±1.31	3.63±1.85	0.038^*^

* statistically significant differences are marked with boldface (Mann-Whitney *U* test). FIM – Functional Independence Measure; NPRS – Numeric Pain Rating Scale; ONLS – Overall Neuropathy Limitations Scale; SD – standard deviation; S-LANSS – Self-Leeds Assessment of Neuropathic Symptoms and Signs Pain Scale

Patients with normal right sural nerve sensory distal latencies (n=11) also had better clinical scale scores across all parameters (Table 7), but the differences from patients with abnormal latencies were significant only for S-LANSS at baseline, FIM at month 12, and ONLS scores at month 12 (p<0.05). In other words, higher sensory distal latency was associated with greater neuropathic pain, reduced functional independence, and greater disability.

**Table 7:** Comparison of clinical scale scores between patients with normal and abnormal sural nerve sensory distal latency values at baseline

**Clinical assessments**	**Sural nerve sensory distal latency values at baseline (mean ± SD)**		**p**
	**Normal (n=11)**	**Abnormal (n=7)**	
NPRS baseline	7.77±1.36	7.86±1.95	0.569
NPRS month 4	8.43±1.51	8.92±1.19	0.457
NPRS month 12	4.31±2.75	4.86±3.34	0.717
S-LANSS baseline	13±4.28	19.43±6.05	0.023^*^
S-LANSS month 4	17.08±3.75	20.71±5.09	0.151
S-LANSS month 12	5±8.77	10±10.41	0.128
FIM baseline	52.14±17.44	47.08±13.89	0.525
FIM month 4	46.29±20.9	31.31±14.14	0.113
FIM month 12	80.57±4.31	69.85±10.33	0.007^*^
ONLS baseline	4.57±1.72	5.38±2.66	0.486
ONLS month 4	7.29±3.82	9±3.14	0.371
ONLS month 12	1.43±0.79	3.23±1.79	0.030^*^

* statistically significant differences are marked with boldface (Mann-Whitney *U* test). FIM – Functional Independence Measure; NPRS – Numeric Pain Rating Scale; ONLS – Overall Neuropathy Limitations Scale; SD – standard deviation; S-LANSS – Self-Leeds Assessment of Neuropathic Symptoms and Signs Pain Scale

## DISCUSSION

Before we proceed with the main findings corroborating the coasting effect, we would like to point out that the electrophysiological findings in our patients are in line with the features of *n*-hexane-induced neuropathy as one of the rare toxic neuropathies with both axonal degeneration and demyelination (32, 33). Baseline measurements show a moderate slowing of conduction velocity and lack of F response as reported earlier (1). London and Albers (14) classified *n*-hexane-induced neuropathy as motor rather than sensorial neuropathy. They stated that the conduction slowing seen in the early stage is due to myelin shrinkage in the nodes of Ranvier in the parts where axonal swelling occurred, which is consistent with the findings in our study.

The coasting effect in *n*-hexane-induced neuropathy and in many other cases of toxic neuropathy (34,35,36) has been well described with EMG and nerve conduction studies (1, 8, 35), but, to the best of our knowledge, ours is the first study to confirm it with both EMG and clinical symptom scales, as NPRS and S-LANSS showed significant changes across the 12-month follow-up. Furthermore, FIM and ONLS scales have shown significant changes in functionality and daily life activities, which worsened at month 4 and improved by the end of the study.

Another aim of our study was to identify early (baseline) EMG parameters that may have some prognostic value for symptom development or worsening that follow. In contrast to earlier assumptions of widespread strong associations, our analysis has demonstrated that significant correlations were mostly limited to conduction velocity parameters, particularly in motor nerves, as demonstrated by significant associations between baseline rMMVel and rUMVel and both functional independence (FIM) and disability severity (ONLS) at month 4. The significant associations between slower baseline motor conduction velocities and lower FIM scores as well as higher ONLS scores suggest that early impairment in impulse propagation may predispose patients to delayed clinical worsening.

Similarly, baseline rCPMVel significantly correlated with FIM scores, further emphasising the prognostic importance of lower extremity motor conduction. In contrast, motor amplitudes and distal latencies did not demonstrate consistent associations with functional scales, suggesting that early functional impairment in *n*-hexane-induced neuropathy may be more closely related to impaired impulse transmission rather than absolute axonal loss.

As for sensory nerve conduction, only rUSDL and rUSVel significantly correlated with NPRS scores, indicating that sensory conduction abnormalities may influence pain perception but do not appear to be major predictors of functional disability. The opposite directions of the correlations observed for rUSDL and rUSVel with NPRS suggest that these associations should be interpreted cautiously and may reflect complex interactions between sensory fibre dysfunction and pain perception rather than a simple ‘more damage – more pain’ relationship.

Importantly, the observed associations between baseline motor conduction velocities and clinical outcomes at month 4 also evidence the coasting effect. Despite cessation of exposure, our patients continued to exhibit clinical deterioration, reflected by the worsening in functional scales at month 4, while electrophysiological abnormalities persisted and only partially improved by month 12. This observation is consistent with the established toxic mechanism of *n*-hexane action, in which accumulation of neurotoxic metabolites results in progressive axonal dysfunction even after exposure has ceased. In this context, baseline slowing of motor nerve conduction velocity may represent an early marker of ongoing neurodegenerative processes, which manifest themselves as functional decline over time. Accordingly, the relationship between early electrophysiological velocity abnormalities and later clinical impairment supports the concept that electrophysiological deterioration may precede and predict the clinical expression of the coasting effect. Conversely, the limited and inconsistent associations observed for amplitudes and distal latencies further suggest that the coasting effect in this study is more closely related to progressive impairment of nerve conduction dynamics rather than abrupt axonal loss, particularly during the early post-exposure period.

### Study limitations

Even though all EMG and clinical scale findings as well as occupational information point to *n*-hexane as the cause of neuropathy, there are no objective data to confirm it, as stated in the participants and methods section. By the time *n*-hexane metabolites were measured, they must have cleared from their blood. Still, we had enough evidence to suspect exposure to *n-*hexane.

The major limitation of our study may be the limited sample size of 18 patients. However, our post hoc power analysis demonstrated adequate statistical power to draw some conclusions. Even so, it limits generalisation and prognosis, and future studies should seek to accumulate information of more patients to run advanced regression or longitudinal models.

Another limitation of this study could be that it was not designed as interventional. We therefore did not account for rehabilitation treatments on the coasting effect as they did not involve a standardised therapy protocol but were tailored according to each patient’s neurological deficits.

## CONCLUSION

Regardless of these limitations, our study points to the real-life consequences of exposure to harmful VOCs in poorly controlled shoe factory environments and establishes the coasting effect through more than one diagnostic parameter. We believe that clinical symptom scales used in our study complement the EMG findings and evidence a meaningful temporal and prognostic association between early motor conduction slowing and delayed functional deterioration characterising the coasting effect. From a preventive perspective, our findings suggest that early electrophysiological evaluation, together with simple and validated clinical scales, may help identify workers at risk of developing neuropathy before irreversible functional impairments occur. Such an approach could inform timely exposure control, medical surveillance, and occupational interventions in high-risk settings. Future studies should be specifically designed to evaluate screening strategies before routine implementation can be recommended.
